# A Genetically Encoded Biosensor for the Detection of Levulinic Acid

**DOI:** 10.4014/jmb.2301.01021

**Published:** 2023-01-27

**Authors:** Tae Hyun Kim, Seung-Gyun Woo, Seong Keun Kim, Byeong Hyeon Yoo, Jonghyeok Shin, Eugene Rha, Soo Jung Kim, Kil Koang Kwon, Hyewon Lee, Haseong Kim, Hee-Taek Kim, Bong-Hyun Sung, Seung-Goo Lee, Dae-Hee Lee

**Affiliations:** 1 Synthetic Biology Research Center, Korea Research Institute of Bioscience and Biotechnology (KRIBB), Daejeon 34141, Republic of Korea; 2 Department of Biosystems and Bioengineering, KRIBB School of Biotechnology, University of Science and Technology (UST), Daejeon 34113, Republic of Korea; 3 Department of Integrative Food, Bioscience and Biotechnology, Chonnam National University, Gwangju 61186, Republic of Korea; 4 Department of Food Science and Technology, Chungnam National University, Daejeon 34134, Republic of Korea; 5 Department of Integrative Biotechnology, College of Biotechnology and Bioengineering, Sungkyunkwan University, Suwon 16419, Republic of Korea

**Keywords:** Levulinic acid, genetically encoded biosensor, transcription factor, *Pseudomonas putida*

## Abstract

Levulinic acid (LA) is a valuable chemical used in fuel additives, fragrances, and polymers. In this study, we proposed possible biosynthetic pathways for LA production from lignin and poly(ethylene terephthalate). We also created a genetically encoded biosensor responsive to LA, which can be used for screening and evolving the LA biosynthesis pathway genes, by employing an LvaR transcriptional regulator of *Pseudomonas putida* KT2440 to express a fluorescent reporter gene. The LvaR regulator senses LA as a cognate ligand. The LA biosensor was first examined in an *Escherichia coli* strain and was found to be non-functional. When the host of the LA biosensor was switched from *E. coli* to *P. putida* KT2440, the LA biosensor showed a linear correlation between fluorescence intensity and LA concentration in the range of 0.156–10 mM LA. In addition, we determined that 0.156 mM LA was the limit of LA detection in *P. putida* KT2440 harboring an LA-responsive biosensor. The maximal fluorescence increase was 12.3-fold in the presence of 10 mM LA compared to that in the absence of LA. The individual cell responses to LA concentrations reflected the population-averaged responses, which enabled high-throughput screening of enzymes and metabolic pathways involved in LA biosynthesis and sustainable production of LA in engineered microbes.

## Introduction

Levulinic acid (LA), a C-5 γ-keto acid, has high potential as a platform chemical, which can be used to produce commercially valuable products, including polycarbonates, plastics, fuel additives, epoxy resins, herbicides, pharmaceuticals, fragrances, and plasticizers [1]. In this context, LA is listed as one of the US Department of Energy’s “top 12 value-added chemicals from biomass” [2]. LA has also been regarded as an alternative to currently used toxic bisphenols or phthalates [1]. The production of hydroxyvalerates and valerolactones from LA [3, 4] has attracted attention because they are widely used as block copolymers [5, 6], advanced fuels [7], and acrylic compounds for drug delivery [8]. The potential market of LA was 2.5 kilotons in 2012 and was expected to expand to 3.8 kilotons by 2020 [9].

LA can be synthesized from lignocellulosic biomass through the Biofine process [10], which is involved in the dehydration of C5- and C6-sugars using acid catalysts from hemicellulose and cellulose, respectively. Recent studies have reported that > 95% of the hemicellulose in lignocellulosic biomass is converted to furfural, which is subsequently converted to furfuryl alcohol in > 95% yield [11, 12], with furfuryl alcohol being rehydrated to LA in approximately 70% yield [13]. Although the Biofine process is a high-yield process that produces LA from lignocellulosic biomass, some challenges remain, including acid catalyst recovery, LA purification, and the high energy and water requirement [14]. In this context, microbial production of LA from lignocellulosic biomass and its conversion to value-added bioproducts may have the potential to overcome the challenges of the Biofine process.

Recent advances in synthetic biology have generated new pathways to produce molecules of interest that are not naturally synthesized by microbes; this includes LA, as the microbial biosynthetic pathways for LA production remain unknown. A recent report designed five biosynthetic pathways to produce LA in engineered *Escherichia coli* and *Saccharomyces cerevisiae* [15]. Among these five designed LA production pathways, two have β-ketoadipate (3-oxoadiptic acid) as an LA precursor. In these pathways, heterologous enzymes encoding β-ketoadipate decarboxylase, acetoacetate decarboxylase, or methyl ketone synthase can convert β-ketoadipate to LA [15]. However, only these enzymes are known to decarboxylate β-ketoadipate to produce LA. Thus, the identification of β-ketoadipate decarboxylase activity and optimization of the assembly of heterologous genes encoding LA production pathway enzymes are the primary bottlenecks to overcome before improving LA production in *E. coli* or *S. cerevisiae*. High-throughput screening (HTS) systems for LA production pathways may address this challenge.

Genetically encoded biosensors (GEBs) have been employed as HTS tools to identify and engineer enzymes and pathways for the biosynthesis of molecules of interest. Among the various types of GEBs, transcription factor (TF)-based genetic circuits have been successfully used to detect industrially valuable molecules, such as L-lysine [16], 3,4-dihydroxy benzoate [17], isoprene [18], and flavonoids [19]. With these GEBs, a fluorescent reporter gene is expressed from a TF-responsive promoter when TFs are induced by molecules of interest. The expression of the fluorescent reporter protein is proportional to the concentration of the molecule. GEBs with a fluorescent reporter can be combined with fluorescence-activated cell sorting (FACS) for HTS at the single-cell level. This GEB-based HTS can markedly reduce the time and labor required to analyze many variant enzymes and pathways [16].

In this study, we presented a GEB for LA sensing. To provide biosynthetic pathways for LA production from renewable resources, including lignin and poly(ethylene terephthalate; PET), we designed biological pathways that can convert the aromatic components of lignin and PET. We then introduced an orthogonal regulatory mechanism into the *E. coli* or *Pseudomonas putida* KT2440 host, which yielded novel GEBs to detect LA. Finally, we probed the LA-sensing ability of the GEB at the single-cell level, which generated the desired input (LA)–output (fluorescence) relationship.

## Materials and Methods

### Bacterial Strains and Culture Conditions

An *E. coli* DH5α strain was obtained from Enzynomics (Korea), *P. putida* KT2440 was purchased from the American Type Culture Collection (ATCC 47054, USA), and *P. putida* KT2440Δ*ttgA* was obtained from Dr. Victor de Lorenzo (CNB-CSIC, Spain). Lysogeny broth (LB) or LB supplemented with an appropriate concentration of ampicillin was used to cultivate the *E. coli* DH5α, *P. putida* KT2440, and *P. putida* KT2440Δ*ttgA* strains. *E. coli* DH5α cells were incubated at 37°C with shaking at 200 rpm. The *P. putida* strains were incubated at 30°C and 200 rpm. To test the GEBs, *P. putida* KT2440 was grown on M9-glycerol broth (1x M9 salts, 0.4% v/v glycerol, 2 mM MgSO_4_, 2.5 ml/l of A9 solution, and 100 μg/ml ampicillin) at 30°C and 200 rpm, while *E. coli* DH5α cells were cultivated on M9-glycerol broth (1x M9 salts, 0.4% v/v glycerol, 2 mM MgSO_4_, 0.1 mM CaCl_2_, and 100 μg/ml ampicillin) supplemented with a trace element solution containing (per liter) 1 g of FeCl_3_·6H_2_O, 0.18 g of ZnSO_4_·7H_2_O, 0.12 g of CuCl_2_·2H_2_O, 0.12 g of MnSO_4_·H_2_O, and 0.18 g of CoCl_2_·6H_2_O. All chemical reagents used in this study were purchased from Sigma-Aldrich (USA).

### Plasmid Construction

The *lvaR* and P_lvaA_ promoters were amplified by PCR using the genomic DNA of *P. putida* KT2440 as a template and the primers 5'-agttcttcacctttgctcatggttctgtaggccctgcctt-3' and 5'-gataacaatttcacacaggatcaattggcagatcgcaagc-3'. Superfolder green fluorescent protein (*sfgfp*) and plasmid backbone were amplified by PCR using the pSEVA131-based CL-GESS plasmid [20] as a template and primers 5'-gcttgcgatctgccaattgatcctgtgtgaaattgttatc-3' and 5'-aaggcagggcctacagaaccatgagcaaaggtgaagaact-3'. PCR was conducted using KODONE DNA polymerase (Toyobo, Japan), and the PCR fragments were assembled using a Gibson Assembly Cloning Kit (New England Biolabs, USA). Primers were obtained from Macrogen (Korea). Sanger sequencing was performed by Macrogen to verify the sequences of the plasmids constructed.

### LA Assay

*E. coli* DH5α harboring LA-GESS v1 was inoculated into 3 ml of LB (+ 100 μg/ml ampicillin) and incubated at 37°C and 200 rpm for 16 h. Then, 3 μl of the culture was transferred to fresh M9 glycerol broth (100 μg/ml ampicillin) and grown at 37°C and 200 rpm for 3 h. A total of 30 μl of the culture broth was transferred to fresh M9 glycerol broth (100 μg/ml ampicillin), and 198 μl of each was loaded onto a black-walled 96-well plate (Greiner Bio-one, Kremsmuster, Austria). Then, 2 μl of the appropriate LA was added to each well and incubated at 37°C and 800 rpm for 22 h using a Thermomixer (Eppendorf, Hamburg, Germany). *P. putida* strains were grown at 30°C in the same way as described above.

### Fluorescence Determination

The signal of the sample incubated for 22 h with the appropriate inducer was determined using a multi-label microplate reader (PerkinElmer, USA) with excitation at 488 nm and emission at 515 nm. Fluorescence at the single-cell level was measured with a FACS Calibur (BD Bioscience, USA) by placing 20 μl of the sample in 1 ml phosphate-buffered saline. For each sample, data for approximately 10,000 events were acquired under forward scatter (FSC), side scatter (SSC), and FL-1 conditions. The cell populations were gated based on the FSC and SSC obtained from wild-type *P. putida* KT2440. Fluorescence data were analyzed using the FlowJo software (Tree Star, USA).

## Results and Discussion

### Design of LA Biosynthesis Pathways Using Monomers of PET and Lignin

**Fig. 1:**
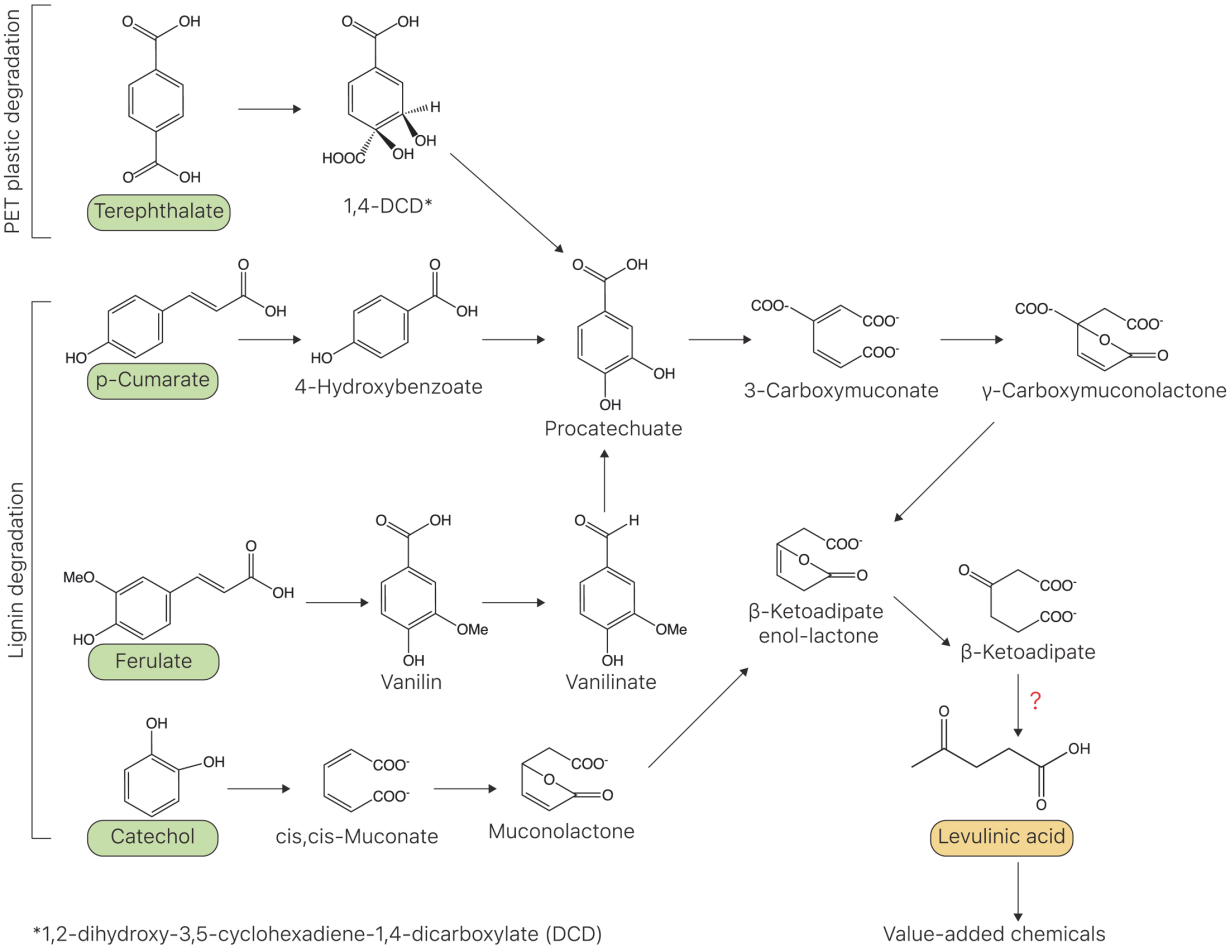
Levulinic acid (LA) biosynthesis pathways using monomers or intermediates of poly(ethylene terephthalate) (PET) and lignin. Terephthalate (TPA), a monomer of PET, was first converted into 1,4-DCD and was subsequently metabolized to procatechuate (PCA). Lignin-derived p-cumarate and ferulate are also converted to PCA. Another lignin-derived catechol can be used to produce LA through cis,cis-muconate and β-ketoadipate. LA produced from these aromatic components of PET and lignin can be further used for producing value-added bioproducts.

LA is produced on an industrial scale from cellulosic biomass at a cost as low as $0.04–$0.10/lb via the Biofine process [21], which produces LA as a substrate for value-added bioproducts. A more environmentally friendly bioprocess to produce LA can be the identification and implementation of a microbe-based metabolic pathway using renewable carbon sources, such as lignin and PET. Although potential biosynthetic pathways of LA have been proposed [15], there are no reports on the production of LA through these designed metabolic pathways in engineered microbes. Instead of using sugars as substrate, we proposed constituent molecules of lignin and PET, including terephthalate (TPA), p-coumarate, ferulate, and catechol. Efficient decomposition approaches for PET and lignin have been developed to valorize lignin and PET. In this context, the monomers or intermediates of these polymers can be considered renewable carbon sources to produce LA in engineered microbes. Recently, PET hydrolases were discovered [22] and engineered to efficiently decompose PET [23]. Once PET is degraded into its monomers, TPA and ethylene glycol are metabolized by the endogenous catabolic pathways of microbes, such as *Ideonella sakaiensis* [22], *Comamonas testosteroni* [24], and *Acinetobacter baylyi* [25]. In these microbes, TPA is converted to protocatechuate (PCA) via the PCA metabolic pathway, and PCA is metabolized to β-ketoadipate, a precursor of LA (Fig. 1). Lignin is the most abundant polymer in nature and accounts for 10–35% of lignocellulosic biomass [26]. Chemical catalyst-based methods and thermochemical methods (pyrolysis and hydrogenolysis) have been developed [27]. Recently, biological delignification has emerged as an eco-friendly and sustainable alternative [28]. Lignin-derived monomers, p-coumarate, ferulate, and catechol have been explored to valorize lignin into value-added chemicals (vanillin, muconate) using engineered microbes [29]. To produce LA from these lignin monomers, p-cumarate and ferulate are converted to PCA and metabolized to β-ketoadipate, whereas catechol is converted to β-ketoadipate via cis,cis-muconate. In these pathways, a decarboxylase enzyme is required to produce LA from β-ketoadipate [15]. Although a few enzymes are known to catalyze this reaction, their activities are insufficient to efficiently convert β-ketoadipate to LA. Therefore, we aimed to design and create a GEB to sense LA to discover novel β-ketoadipate decarboxylase and engineer known enzymes that produce LA from β-ketoadipate.

### Genetically Encoded Biosensors to Detect LA in *E. coli*

**Fig. 2:**
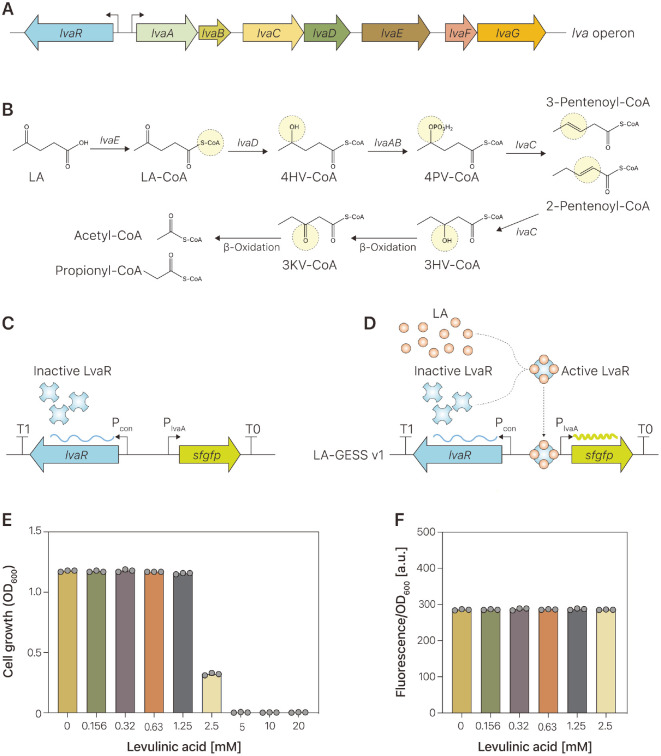
Design and development of an LA-responsive biosensor. (**A**) Organization of the *lvaR*ABCDEFG operon responsible for LA metabolism in *Pseudomonas putida* KT2440. (**B**) Metabolic pathway for LA metabolism encoded by *lvaR*ABCDEFG operon. LA, levulinic acid; 4HV, 4-hydroxyvalerate; 3HV, 3-hydroxyvalerate; CoA, coenzyme-A; 4PV-CoA, 4- phosphovaleryl-CoA; 3KV-CoA, 3-ketovaleryl-CoA. (**C**) Organization of LA-responsive biosensor. The genes (*lvaR*, *sfgfp*), promoters (P_con_, P_lvaA_), and terminators (T1, T0) are indicated by thick arrows, bent arrows, and stem-loops, respectively. *lvaR* of *P. putida* KT2440 was expressed by the P_con_ promoter, and the P_lvaA_ promoter regulated the expression of *sfgfp*. LvaR proteins are inactive in the absence of their effectors. (**D**) Proposed mechanism for the LA-responsive biosensor. LvaR binding to its binding site, which is located upstream of the P_lvaA_ promoter, is triggered by LA (orange), which leads to the expression of the reporter gene, *sfgfp*. (**E**) Effect of LA on *E. coli* DH5α cell growth. Various amounts of LA ranging from 0 to 20 mM were added to the culture broth, and the cells were grown at 37°C. (**F**) Responses of LA-responsive biosensor to various amounts of LA. Green fluorescence of sfGFP was normalized by cell growth at 600 nm (OD_600_).

To create a GEB that responds to LA, a literature survey was conducted to identify a TF that can recognize LA as a cognate ligand. Recently, a metabolic pathway for catabolizing LA in *P. putida* KT2440 was reported [30]. Polycistronic genes, designated as the *lvaABCDEFG* operon, were found in transposon mutant libraries. Upstream of this operon, the *lvaR* gene is oriented divergently from the operon (Fig. 2A). This *lvaR* gene is a TF with a σ54 interaction domain and is homologous to the propionate metabolism activator *prpR*. The *lvaR* gene regulates the *lva* operon [30]. In the presence of LA, LvaR activates the expression of the *lva* operon, which is responsible for LA metabolism in the following pathway: LA is activated as a coenzyme A-thioester, levulinyl-CoA (LA-CoA), and LA-CoA is subsequently reduced to 4-hydroxyvaleryl-CoA (4HV-CoA). 4HV-CoA is phosphorylated at the γ-position to yield 4-phosphovaleryl-CoA (4PV-CoA), and 4PV-CoA is dephosphorylated to form a pentenoyl-CoA species (probably 3-pentenoyl-CoA). Pentenoyl-CoA is hydrated to produce 3-hydroxyvaleryl-CoA (3HV-CoA), which can be further oxidized via β-oxidation to yield acetyl-CoA and propionyl-CoA (Fig. 2B).

We took advantage of a native regulatory system to enable the GEB to convert the intracellular LA concentration into a fluorescent output. A single plasmid, pLA-GESS1, was generated to encode the *lvaR* regulator and the *sfgfp* gene. The expression of the *lvaR* gene was under the control of the constitutive promoter in the direction opposite to the transcription of *sfgfp*, while *sfgfp* transcription was regulated by the *lvaR*-responsive promoter P_lvaA_ (Fig. 2C). The expression of *sfgfp* was expected to be regulated by LA alone inside the *E. coli* cells because *E. coli* cannot metabolize LA or produce LA from other carbon sources. T1 and T0 terminators were used to terminate the transcription of *lvaR* and *sfgfp*, respectively. The pSEVA131 backbone plasmid was derived from a commonly used medium-copy plasmid that employs a pBBR1 replication origin. The LvaR protein is presumably nonfunctional in the absence of LA, whereas LvaR binds to the P_lvaA_ promoter and leads to *sfgfp* expression in the presence of LA (Fig. 2D). In this study, LvaR served as a transcriptional regulator to detect the presence of LA and induce the expression of a ﬂuorescent reporter protein. To monitor the in vivo LA concentration in *E. coli*, we introduced a pLA-GESS1 plasmid encoding an LA-responsive GEB into *E. coli* DH5α cells. Transformed cells were grown in M9 minimal medium supplemented with different concentrations of LA (0–20 mM). The cell growth and fluorescence responses were measured (Figs. 2E and 2F). As expected, 2.5–20 mM LA was toxic to *E. coli* cell growth. *E. coli*-pLA-GESS1 cells grown in the presence of 2.5 mM LA showed a 73% decrease in cell growth compared to *E. coli* in the absence or presence of LA (0.156–1.25 mM; Fig. 2E). LA concentrations ranging from 5 mM to 20 mM showed no *E. coli*-pLA-GESS1 cell growth. *E. coli*-pLA-GESS1 cells showed no response to any of the tested LA concentrations, as analyzed by fluorometry and flow cytometry, which made it difficult to correctly determine the ratio between the maximum induced signal and uninduced signal (Fig. 2F). To build the LA-responsive genetic circuit, we used transcriptional regulation of *P. putida* KT2440 and tested its performance in *E. coli*. GEBs rely on bioparts within the central dogma of the host to produce the necessary components, which can reduce host fitness by sharing essential cellular resources [31]. Therefore, the performance of LA-responsive genetic circuits is limited by the incompatibility of components in *E. coli* DH5α cells, such as crosstalk and toxicity [32]. Since we could not observe growth inhibition of *E. coli* DH5α cells containing the LA-responsive genetic circuit, crosstalk for non-functionality of the LA-responsive genetic circuit may be caused by the native TFs of *E. coli* DH5α cells. In addition, true ligands of LvaR TF should be considered because *P. putida* KT2440 can metabolize LA whereas the *E. coli* DH5α strain was not known to utilize it, which suggested that intermediates of LA degradation pathways in *P. putida* KT2440 can be a true inducer for LavR activation.

### Genetically Encoded Biosensors to Detect LA in *P. putida* KT2440

**Fig. 3:**
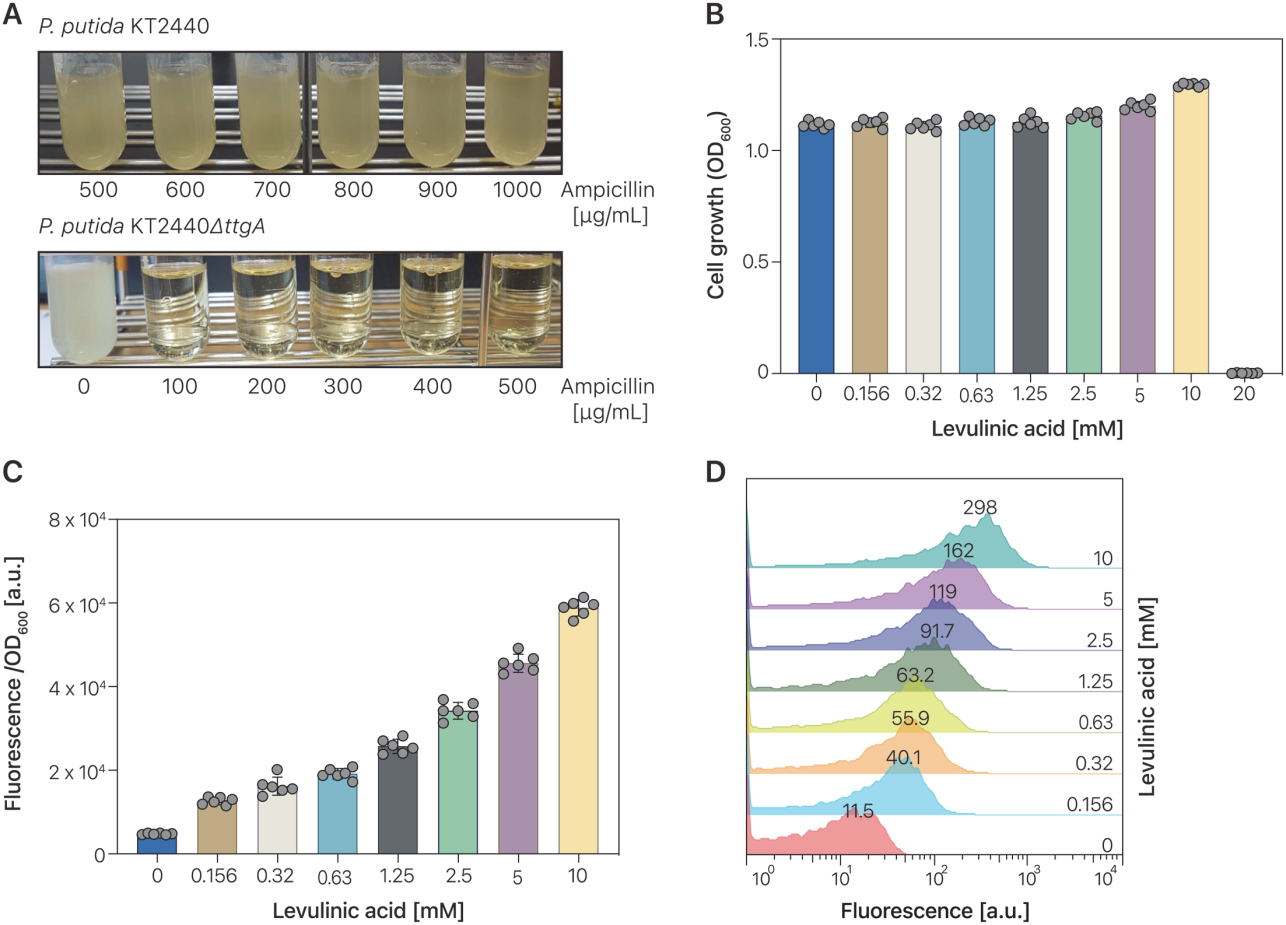
LA-responsive biosensors in *Pseudomonas putida* KT2440. (**A**) Ampicillin resistance of wild-type *P. putida* KT2440 and *P. putida* KT2440Δ*ttgA* mutant. (**B**) Effect of LA on *P. putida* KT2440Δ*ttgA* mutant cell growth. Various LA amounts ranging from 0 to 20 mM were added to the culture broth, and the cells were grown at 30°C. (**C**) Responses of LAresponsive biosensors to various amounts of LA in the *P. putida* KT2440Δ*ttgA* mutant at the population level. Green fluorescence of sfGFP was determined using a fluorometer and normalized by cell growth at 600 nm (OD_600_). (**D**) Fluorescence of LA-responsive biosensor at the single cell level. Fluorescence was induced by adding different concentrations of LA and determined by fluorescence-activated cell sorting (FACS). FACS-generated histograms from one representative experiment out of three are presented.

The soil bacterium and platform strain *P. putida* KT2440 is a model host for metabolic engineering that can harbor reactions requiring toxic substrates and products that practically no other bacterial host can tackle [33]. We focused our attention on this bacterium to probe the performance of the LA-responsive GEB because the LvaR TF and its responsive promoter, P_lvaA_, are from *P. putida* KT2440. To this end, we introduced the pLA-GESS1 plasmid, which is derived from the pSEVA131 plasmid and is compatible with *P. putida* KT2440, into the *P. putida* KT2440 strain, yielding the *P. putida* KT2440-pLA-GESS1 strain. The biosensor strain was grown in M9 minimal medium supplemented with different concentrations of LA (0–20 mM). The *P. putida* KT2440-pLA-GESS1 strain showed almost no response to all the tested concentrations of LA. We found that the *P. putida* KT2440-pLA-GESS1 strain was ampicillin-resistant (Fig. 3A, Top panel). Since the efflux pump encoded by the *ttgA*BC gene sufficed to endow *P. putida* KT2440 with a high level of tolerance to β-lactams [34], we switched wild-type *P. putida* KT2440 with the *P. putida* KT2440Δ*ttgA* strain to test the LA biosensor. The *P. putida* KT2440Δ*ttgA*-pLA-GESS1 strain showed sensitivity to ampicillin (Fig. 3A, Bottom panel) and similar cell growth (0.156–10 mM LA) to that in the absence of LA (Fig. 3B). However, this strain did not grow in the presence of 20 mM LA. The ﬂuorescence response to various LA concentrations (0–10 mM) was examined at the population level using *P. putida* KT2440Δ*ttgA*-pLA-GESS1 (Fig. 3C). A linear dependence between LA concentration and sfGFP ﬂuorescence was observed in the range of 0.156–10 mM, with 0.156 mM LA being the limit of LA detection in this condition. The maximal ﬂuorescence increase was 12.3-fold when *P. putida* KT2440Δ*ttgA*-pLA-GESS1 cells were exposed to exogenous 10 mM LA. Some biosensors show bimodal or heterogeneous induction of ﬂuorescence [35, 36], which limits their applicability to the HTS of enzymes and metabolic pathways. To examine the ﬂorescence induction pattern of pLA-GESS1, ﬂuorescence intensity was compared using ﬂow cytometry (Fig. 3D) with that using a ﬂuorometer (Fig. 3C). The pLA-GESS1 showed LA concentration-dependent fluorescence at the single cell level, suggesting that the individual cell fluorescence mirrored the population-averaged response to LA concentration determined by the fluorometer. In addition, the results showed that the pLA-GESS1 biosensor required at least 0.156 mM LA to detect LA and exhibited a linear relationship between ﬂuorescence and LA concentration at concentrations up to 10 mM (Fig. 3D).

## Conclusions

In this study, we developed a GEB for detecting LA, which has a high potential as a platform chemical that can be used to produce commercially valuable products. The LavR transcription factor was adopted from the soil bacterium *P. putida* KT2440 and was heterologously expressed in *E. coli* or *P. putida* KT2440. The LA-responsive biosensor showed no response to exogenous LA in *E. coli* DH5α cells, whereas it exhibited a very strong response and wide detection range to LA in *P. putida* KT2440Δ*ttgA*. This LA-responsive biosensor can be applied to identify novel enzymes and pathways involved in LA production, thereby helping in the development of microbial cell factories for LA production.
